# Temporal comorbidity patterns preceding MASLD-related major adverse liver outcomes: a nationwide population-based case–control study in Sweden

**DOI:** 10.1136/bmjph-2025-003322

**Published:** 2025-11-13

**Authors:** Joost Boeckmans, Linnea Widman, Rickard Strandberg, Ying Shang, Axel Wester, Hannes Hagström

**Affiliations:** 1 Department of Medicine, Karolinska Institutet, Stockholm, Sweden; 2 In Vitro Liver Disease Modelling Team, Department of In Vitro Toxicology and Dermato-Cosmetology, Faculty of Medicine and Pharmacy, Vrije Universiteit Brussel, Brussels, Belgium; 3 Department of Upper GI, Karolinska University Hospital, Stockholm, Sweden

**Keywords:** Epidemiology, Preventive Medicine, Population Surveillance, Community Health

## Abstract

**Background:**

The patterns of comorbidities preceding major adverse liver outcomes (MALOs) in metabolic dysfunction-associated steatotic liver disease (MASLD) are unclear.

**Methods:**

All patients from the Swedish National Patient Register with MASLD-related MALO between 2006 and 2022 were identified, and matched for age, sex and diagnosis year, with individuals having MASLD without MALO, and population controls. Hierarchical clustering using International Classification of Diseases-10 codes was employed for identifying comorbidities, with comparison between the groups. Median times of comorbidities until MALO were calculated.

**Results:**

996 patients with MASLD-related MALO and median age of 67 years (IQR=60–73), of which 49% men, were matched with 3921 individuals with MASLD without MALO and 9056 population controls. A median of nine comorbidity categories was observed in patients with MALO, who exhibited a cardiometabolic cluster with type 2 diabetes (49%) and hypertension (56%), which were diagnosed 6 years before MALO. Heart failure and atrial fibrillation/flutter preceded MALO in 13% and 14% and were diagnosed 4 years beforehand. Myocardial infarction occurred 6 years prior to MALO in 8%, while angina pectoris developed 8 years prior in 14% of patients. Sleep disorders were seen 8 years before MALO in 14% of patients. All identified comorbidities were present at lower rates in individuals with MASLD without MALO and population controls (p<0.001).

**Conclusions:**

Patients with MASLD-related MALO are frequently multimorbid. Cardiac disease and sleep disorders, with cardiometabolic risk factors, characterise individuals developing MALO. These patterns may simplify case finding and earlier interventions.

**WHAT IS ALREADY KNOWN ON THIS TOPIC**

- Major adverse liver outcomes (MALOs), including cirrhosis and its complications, are life-changing events in people living with metabolic dysfunction-associated steatotic liver disease (MASLD), and a major public health issue. Specific comorbidity patterns in people with MASLD developing MALOs could guide case finding strategies.
- Multiple clinical and epidemiological studies reported a high prevalence of metabolic risk factors in patients with MASLD-related MALO, including type 2 diabetes mellitus (~50%) and obesity (~40%). Further, several studies described a high prevalence of cardiovascular disease. However, no studies are available describing the natural history of comorbidities prior to MASLD-related MALO in an untargeted way with temporal occurrence and comparison with matched comparators.

**WHAT THIS STUDY ADDS**

- This nationwide population-based case-control study was designed to map the natural history of comorbidities preceding a MASLD-related MALO using an untargeted hierarchical clustering approach employing International Classification of Diseases codes.
- Patients who developed MASLD-related MALO were multimorbid and defined by cardiometabolic risk factors in conjunction with sleep disorders and different cardiac diseases, including heart failure, atrial fibrillation/flutter and ischaemic heart disease. These comorbidities occurred 4–8 years before the development of MALO, paving avenues for case finding strategies, intensified liver monitoring and preventive measures.

**HOW THIS STUDY MIGHT AFFECT RESEARCH, PRACTICE OR POLICY**

- Patients with MASLD who develop a MALO frequently have cardiometabolic risk factors, cardiac disease, and sleep disorders, which could be used in case finding strategies to take preventive measures targeting both metabolic liver and cardiovascular disease, including but not limited to physical exercise and glucagon-like peptide-1 receptor agonists.

## Introduction

 Metabolic dysfunction-associated steatotic liver disease (MASLD) affects ~38% of the adult population and represents a spectrum of disease stages ranging from isolated liver steatosis to progressive metabolic dysfunction-associated steatohepatitis (MASH), cirrhosis and hepatocellular carcinoma (HCC).^1 2^ MASLD is associated with a reduced life expectancy,^3^ particularly on developing liver cirrhosis and HCC.^4^ It is estimated that patients with compensated cirrhosis have a life expectancy of 12 years or more, compared with approximately 2 years for patients with decompensated cirrhosis.^5^ Early detection of patients at risk of progressive MASLD is, therefore, crucial to prevent end-stage liver disease and subsequent premature death.^6^ Much effort has been made to establish screening modalities for detecting MASLD-related advanced fibrosis in high-risk populations using non-invasive tests, for instance in patients with type 2 diabetes mellitus (T2DM).^7^ Although such screening strategies aim to find MASLD/MASH at a relatively early stage, up to 10% of individuals with MASLD in a high-risk setting may already have cirrhosis,^8^ where the HCC incidence rate is around 11 per 1000 person years.^9^ Furthermore, ~40% of patients with cirrhosis are diagnosed first on developing hepatic decompensation,^10^ emphasising the need for optimised strategies allowing earlier identification, for example, through case finding based on prior medical conditions.

Comorbidities that are frequently associated with MASLD are, apart from T2DM and obesity,^2^ heart failure,^11^ coronary heart disease, chronic kidney disease, obstructive sleep apnoea and gallstone disease,^12^ which substantiates the relevance of composing clinical profiles for case finding of patients with undiagnosed MASLD who will progress to major adverse liver outcome (MALO). Further, although cardiac-specific mortality largely transcends liver-specific mortality in individuals with MASLD (mortality rate per 1000 person years of 5.54), liver-specific death still accounts for a mortality rate of 1.75 per 1000 person years.^13^

No pharmacological treatment is approved for MASLD cirrhosis, while the first drug treatment option for precirrhotic disease, resmetirom (a thyroid hormone receptor-β agonist), is now available in the USA, which justifies targeted screening for these patients. In addition, multiple other compounds are in late clinical development for the treatment of patients with precirrhotic MASH, including glucagon-like peptide-1 (GLP-1) receptor agonists.^14^

The natural history of comorbidities preceding a MASLD-related MALO remains vague, and it is unknown which medical conditions cluster together in individuals who develop MALO, and when these appear. Better knowledge of temporal patterns of comorbidities prior to MALO can offer opportunities for setting up case finding strategies and initiating interventions before cirrhosis and subsequent complications occur. We hypothesised that distinct comorbidity phenotypes can be identified via hierarchical clustering of International Classification of Diseases (ICD) codes that precede MALO in patients with MASLD.

## Study participants and methods

### Cohort

The DEcoding the epidemiology of LIVER disease in Sweden (DELIVER) cohort was used to identify all patients in Sweden with MASLD-related MALO and matched individuals with MASLD without MALO or general population controls without MASLD or MALO. The DELIVER cohort is based on ICD codes from the National Patient Register between 1964 and 2022.^15^ The Personal Identification Number allows linking the DELIVER cohort to additional Swedish registers to ascertain information on, for example, the dates of birth and death, country of birth (total population register), causes of death (cause of death register), sociodemographic factors (longitudinal integrated database for health insurance and labour market studies), dispensed medications since 1 July 2005 (prescribed drug register) and malignancies (cancer register). Data were obtained from both in- and outpatient visits and capturing both main and secondary diagnoses. The ‘‘Strengthening the Reporting of Observational Studies in Epidemiology’’ (STROBE) checklist is provided as a supplement.

### Definitions of patients with MASLD-related MALO and comparators

The study period began on 1 January 2006 and ended on 31 December 2022. This allowed for at least 5 years to ascertain pre-existing comorbidities in the ICD-10 era with the coverage of outpatient care since 2001 and inpatient care and daytime surgery since 1997. A graphical overview of the study design is provided in online supplemental figure 1.

MALO because of MASLD was defined as having at least one ICD-10 code from online supplemental table 1, together with recording of K76.0 (ie*,* MASLD) or K75.8 (ie*,* MASH) before or within 3 months after the respective ICD-10 code of MALO in the DELIVER cohort, and cause of death register.

At the time of first diagnosis of a MASLD-related MALO, each case was matched with up to four individuals with MASLD without MALO, classified as ‘MASLD’/‘MASH’, and with up to ten general population controls without MASLD or MALO. Individuals were matched on age, sex and year of MALO diagnosis. A matching window of 2 years before and after MALO diagnosis was applied for individuals with MASLD without MALO to increase the sample size of this control group.

Baseline for patients with MASLD-related MALO was defined as the date of first coding for MALO for outpatients or the date of discharge for inpatients. Baseline for population controls was the date of matching with a patient with MASLD-related MALO. Baseline for patients with MASLD without MALO was the date of matching belonging to these individuals.

Exclusion criteria for cases (before matching) were MALO (any ICD-10 code in online supplemental table 1) prior to 1 January 2006, having another liver disease or competing aetiologies other than MASLD that predispose to liver disease (online supplemental table 1) at or prior to the diagnosis of MALO or up to 90 days afterwards (this is an ascertainment period to apply the exclusion criteria with more certainty), and death not related to MALO during the 90-days ascertainment period.

Exclusion criteria for patients with MASLD without MALO and population controls were MALO (any ICD-10 code in online supplemental table 1) prior to the end of the 90-days ascertainment period (back to 1997), having an exclusion criterion from online supplemental table 2 prior to the end of the 90-days ascertainment period, and dead during the 90-days ascertainment period. Exclusion criteria that applied for all study participants were emigration prior to the end of the 90-days ascertainment period, age under 18 years and administrative reasons (reused or wrongly coded personal identity number, no information on country of birth, civil status or education). Individuals with MASLD-related MALO without matched comparator were excluded.

### Outcomes

The primary outcome was defined as clinically relevant comorbidities that were present at or prior to the time of diagnosis of MASLD-related MALO. Secondary outcomes were the prevalence of the identified comorbidities in patients with MASLD-related MALO compared with individuals with MASLD without MALO and population controls, and the median times between first coding of the comorbidity and MALO occurrence.

### Statistical analyses

Continuous data were reported as medians with IQRs and categorical data were reported as frequencies with percentages. A Wilcoxon rank sum test or Pearson’s χ^2^ test was used as appropriate.

First, ICD-10 categories (eg*,* ‘hypertensive diseases; I10–I15’) were screened to identify diseases and conditions that potentially relate to MASLD-related MALOs. This applied for the first coding of unique ICD-10 codes prior to baseline. A study participant could only score 1 on each category (eg, if someone had essential hypertension (I10) and hypertensive renal disease (I12), then it counted as 1 since these codes are both in the I10–I15 category). To avoid artificial clustering processes, ICD-10 categories that included a code used to define the cases were excluded (eg*,* K74.6 was included in the case definition, so K70–K77 was excluded), as well as Z-codes that mainly include unspecific codes related to contact with healthcare, R-codes that mainly include unspecific symptoms and signs, T-codes that include complications of medical care and sex-specific N-codes. ICD-10 categories with an occurrence of at least 5% in the MASLD-MALO group were considered as relevant and selected for hierarchical clustering analysis using ICD-10 codes on the three-digit level (eg, A00). Second, hierarchical clustering (R-package-*Hmisc*, function-*varclus* and similarity-*bothpos*) was performed for the proportional occurrence of ICD-10 codes. The main cluster was selected from the cases with MALO and compared with clusters in the MASLD without MALO individuals and population controls, selected by the most frequently occurring comorbidities in the cases with MALO. Median times between first coding for clinically relevant comorbidities and baseline for cases with MASLD-related MALO were calculated and presented on a timeline. Missing data were considered of minor impact because of the register-based approach. Analyses were performed using R V.4.4.1 (2024-06-14 ucrt)—‘Race for Your Life’ and GraphPad_Prism_version_8.4.3.

### Patient and public involvement statement

Patients were not involved in the study design or conduct because of the register-based approach. Results cannot be directly disseminated to the study participants because of study participant anonymity when using Swedish national healthcare registers.

## Results

### Cohort characteristics

**Figure 1:**
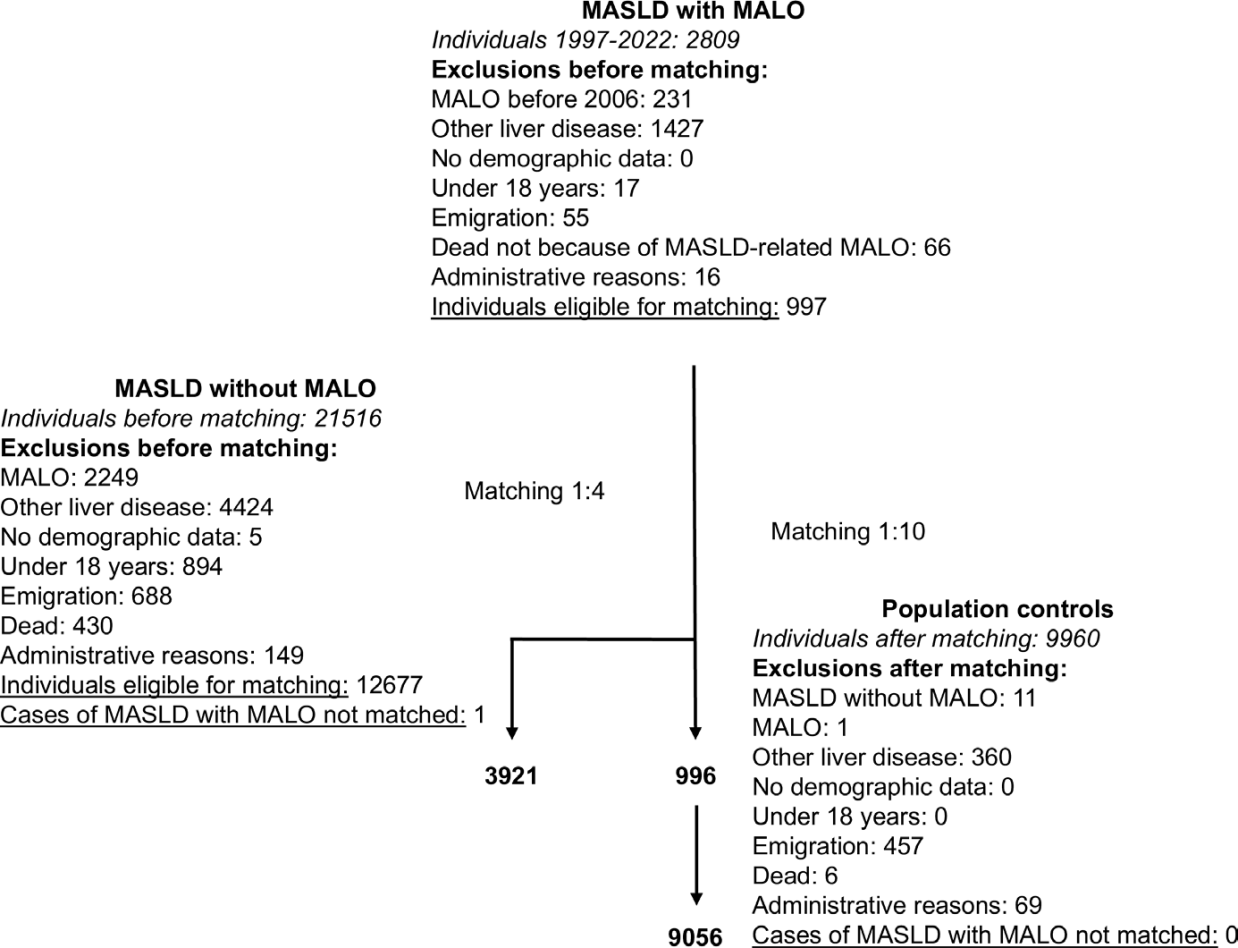
Flowchart showing exclusions and matching of patients with MASLD-related MALO to individuals with MASLD without MALO and population controls. MALO, major adverse liver outcome; MASLD, metabolic dysfunction-associated steatotic liver disease.

There were 997 eligible patients with MASLD-related MALO identified between 2006 and 2022, of which 996 individuals were matched (up to 1:4) with 3921 people living with MASLD without MALO, and with 9056 people from the general population (up to 1:10) (figure 1).

Patients with MALO were mainly defined by compensated cirrhosis (n=651), followed by decompensated cirrhosis (n=249), HCC (n=93) and both decompensated cirrhosis and HCC (n=1). Two individuals had a liver transplantation as the first recorded MALO.

Patients with MALO were in median 67 years (IQR=60–73), and 49% were men. The included number of patients with MALO increased during the study period, with the lowest number included in the first calendar period of inclusion (13% between 2006 and 2010) and the highest number included in the final calendar period (39% between 2019 and 2022). Patients with MALO had the most healthcare contacts in the year preceding baseline, with a median of 4 (IQR=2–8) visits compared with 3 (IQR=1–6) for individuals with MASLD without MALO and 0 (IQR=0–2) for population controls (all p<0.001) (table 1). Corresponding mean numbers of healthcare contacts were 6 (SD=7), 4 (SD=5) and 2 (SD=4).

**Table 1:** Study participants characteristics

Parameter	MASLD with MALO	MASLD without MALO	Population controls	P value (MASLD with MALO vs without MALO)	P value (MASLD with MALO vs population controls)
Included persons	996	3921	9056		
Age (years)	67 (60–73)	66 (59–73)	67 (60–73)	0.094	0.8
Sex (male)	490 (49%)	1924 (49%)	4405 (49%)	>0.9	0.7
Year of inclusion				0.7	>0.9
2004–2005 (matching within 2 years for MASLD without MALO)	0 (0%)	55 (1%)	0 (0%)		
2006–2010	126 (13%)	477 (12%)	1175 (13%)		
2011–2014	193 (19%)	781 (20%)	1768 (20%)		
2015–2018	285 (29%)	1153 (29%)	2608 (29%)		
2019–2022	392 (39%)	1455 (37%)	3505 (39%)		
Education				0.045	<0.001
<10 years	276 (28%)	1007 (26%)	2072 (23%)		
10–12 years	497 (50%)	1888 (48%)	3991 (44%)		
>12 years	223 (22%)	1026 (26%)	2993 (33%)		
Country of birth				0.2	<0.001
Sweden	788 (79%)	3201 (82%)	7892 (87%)		
Nordic except Sweden	43 (4.3%)	175 (4.5%)	354 (3.9%)		
European except Nordic	76 (7.6%)	241 (6.1%)	436 (4.8%)		
Other and unknown	89 (8.9%)	304 (7.8%)	374 (4.1%)		
Marital status				0.086	<0.001
Married	503 (51%)	2142 (55%)	5105 (56%)		
Unmarried	186 (19%)	665 (17%)	1487 (16%)		
Widow	87 (8.7%)	350 (8.9%)	843 (9.3%)		
Divorced	220 (22%)	764 (19%)	1621 (18%)		
Healthcare characteristics					
Number of healthcare contacts in the year preceding baseline	4 (2–8)	3 (1–6)	0 (0–2)	<0.001	<0.001

Data presented as medians with IQRs (continuous variables) or absolute and relative frequencies (categorical variables).

MALO, major adverse liver outcome; MASLD, metabolic dysfunction-associated steatotic liver disease.

### Screening of ICD-10 codes

Screening of ICD-10 categories resulted in 64 unique categories with an occurrence of at least 5% in the sample of MASLD-related MALO cases. Patients with MASLD-related MALO had more diagnoses grouped by ICD-10 categories (median of nine categories; IQR=5–13) compared with individuals with MASLD without MALO (median of seven categories; IQR=4–11, p<0.001) and population controls (median of four categories; IQR=2–7, p<0.001). Corresponding mean numbers of ICD-10 categories were 10 (SD=6), 8 (SD=5) and 5 (SD=4).

**Figure 2:**
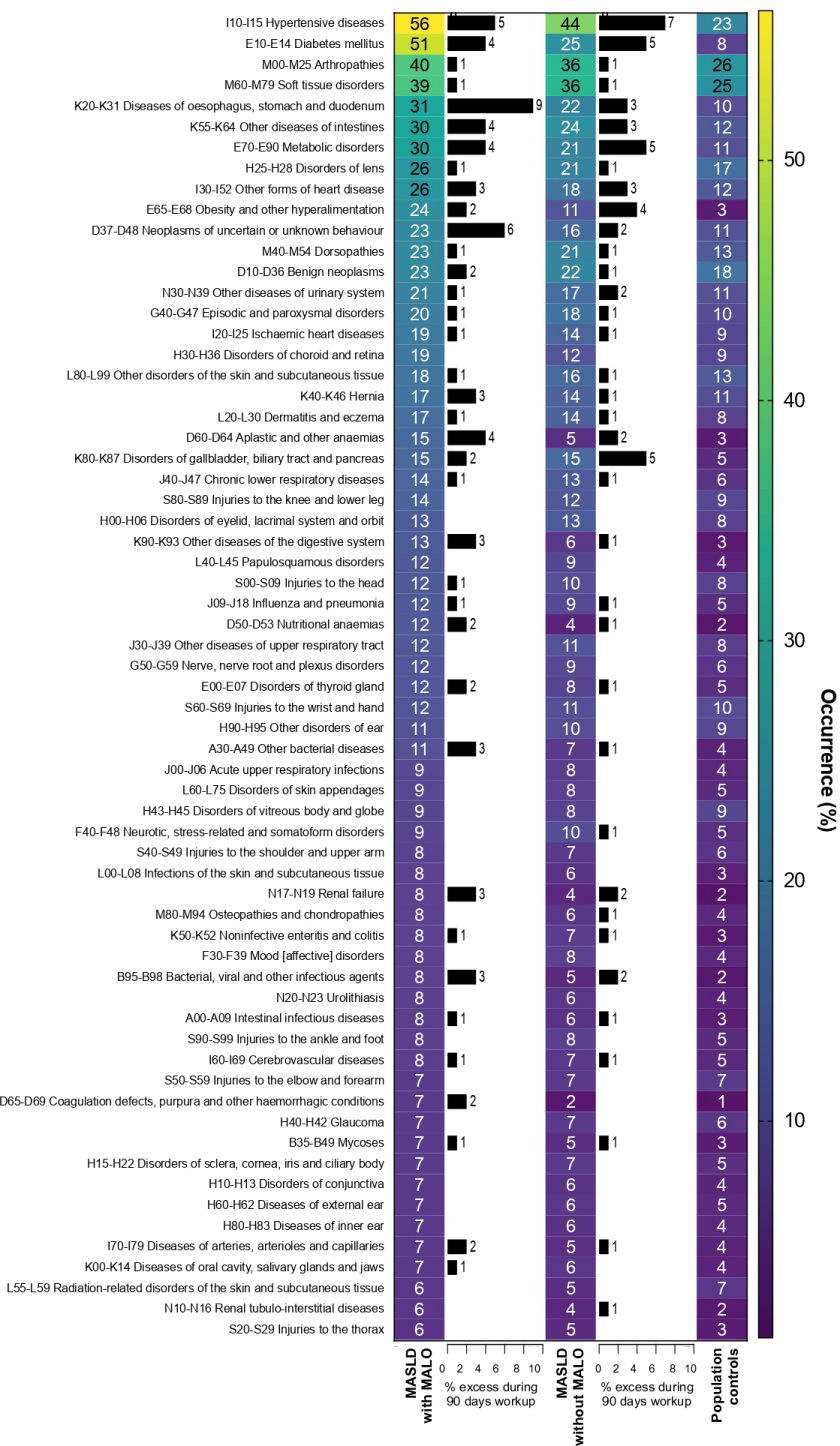
Occurrence of ICD-10 categories in patients with MASLD-related MALO and matched individuals with MASLD without MALO and population controls. A cut-off of 5% occurrence was applied to the ICD-10 categories in patients with MASLD-related MALO, and the categories were sorted by highest to lowest occurrence based on the patients with MASLD-related MALO. Diagnoses of comorbidities made in the 90 days after the first diagnosis of MASLD-related MALO or MASLD without MALO are shown with black bars. ICD, International Classification of Diseases; MALO, major adverse liver outcome; MASLD, metabolic dysfunction-associated steatotic liver disease.

The most frequently occurring comorbidity categories in patients with MALO were ‘hypertensive diseases’ (56%), ‘diabetes mellitus’ (51%) and ‘arthropathies’ (40%) (figure 2). ‘Hypertensive diseases’ were also the dominating comorbidity category in the matched patients with MASLD without MALO (44%), followed by ‘arthropathies’ and ‘soft tissue disorders’ (both 36%) (online supplemental figure 2). In the matched comparators of the general population, ‘arthropathies’ occurred most frequently (26%), while ‘soft tissue disorders’ (25%) and ‘hypertensive diseases’ (23%) were the second and third most frequent ICD-10 categories (online supplemental figure 3). The difference in occurrence of ICD-10 categories between patients with MASLD-related MALO and individuals with MASLD without MALO and population controls was most pronounced for ‘diabetes mellitus’, with absolute differences of 26% and 43%, respectively. Other categories that were more common in patients with MASLD-related MALO than in individuals with MASLD without MALO and population controls were ‘obesity and hyperalimentation’ (differences of 13% and 21%, respectively) and ‘hypertensive diseases’ (differences of 12% and 33%, respectively) (online supplemental figure 4). Further, during the first 90 days after experiencing a first MASLD-related MALO, an additional 4% of all patients were diagnosed with diabetes mellitus and 5% of patients were diagnosed with a hypertensive disease (figure 2).

### Hierarchical clustering of ICD-10 codes

**Figure 3:**
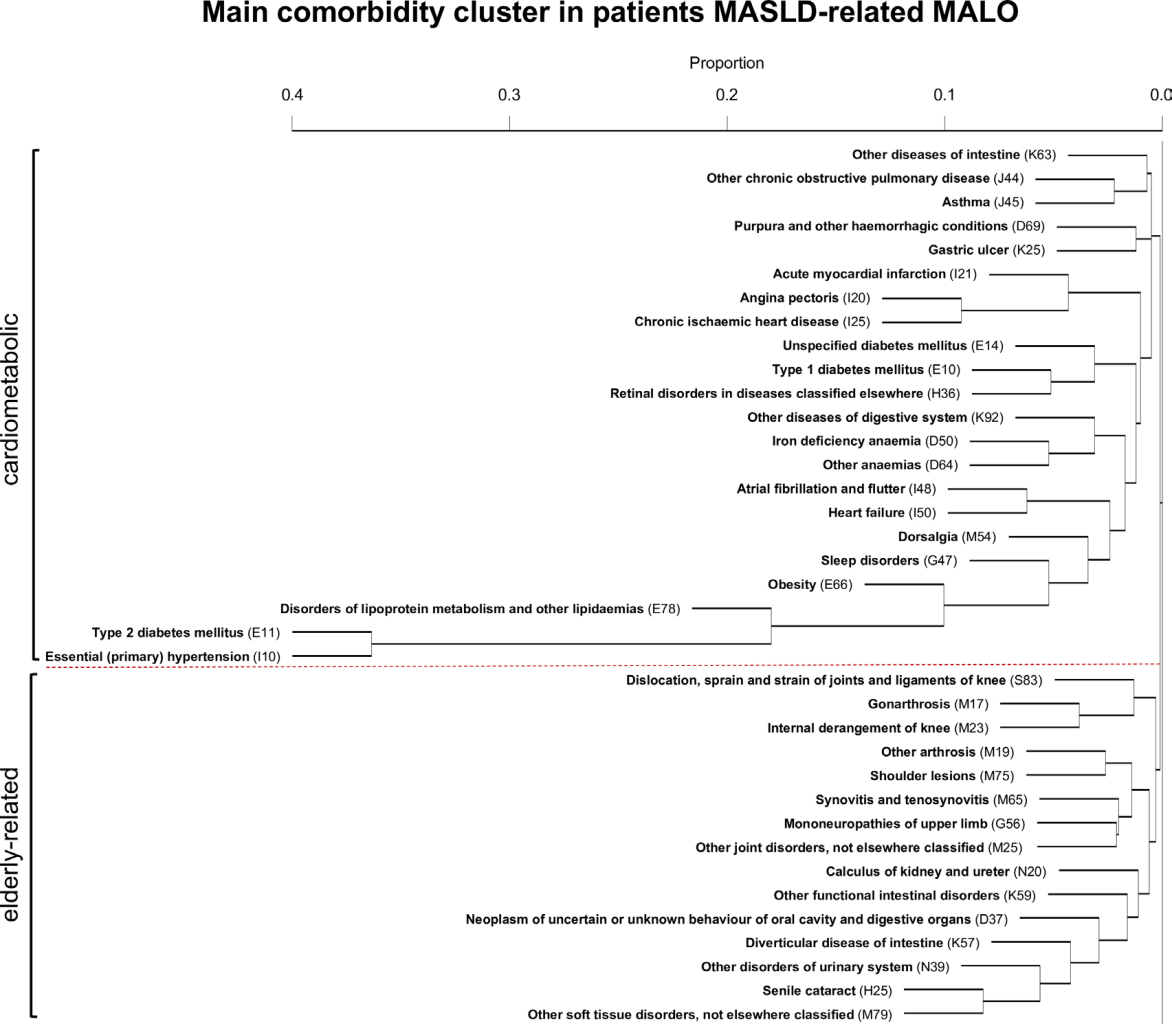
Main comorbidity cluster for patients with MASLD-related MALO. A primary cardiometabolic and secondary elderly-related cluster within the main comorbidity cluster is separated by the dotted red line. MALO, major adverse liver outcome; MASLD, metabolic dysfunction-associated steatotic liver disease.

Hierarchical clustering was done using all three-digit ICD-10 codes (eg*,* I10) included in the 64 identified ICD-10 categories (online supplemental table 3). In patients with MASLD-related MALO, one main cluster was identified including a primary and a secondary subcluster (the full clusters are provided as supplementary files: onlinesupplemental annex 1^3^). The primary subcluster consisted mainly of cardiometabolic conditions and was dominated by the concurrence of ‘T2DM’ and ‘essential hypertension’. Associated conditions were ‘disorders of lipoprotein metabolism and other lipidaemias’, ‘obesity’, ‘sleep disorders’, ‘dorsalgia’, ‘heart failure’, ‘atrial fibrillation and flutter’ and different anaemias. The secondary subcluster generally consisted of elderly-related conditions, including ‘senile cataract’ and arthrosis- and joint-related conditions (figure 3). In the matched patients with MASLD without MALO, a similar cardiometabolic cluster was observed, with a closer association of T2DM and essential hypertension with coronary artery disease (ie*,* chronic ischaemic heart disease, angina pectoris and acute myocardial infarction) (online supplemental figure 5A). There was no cardiometabolic cluster originating from T2DM in the matched population controls (online supplemental figure 5B).

### Timeline of comorbidities preceding a MASLD-related MALO

**Figure 4:**
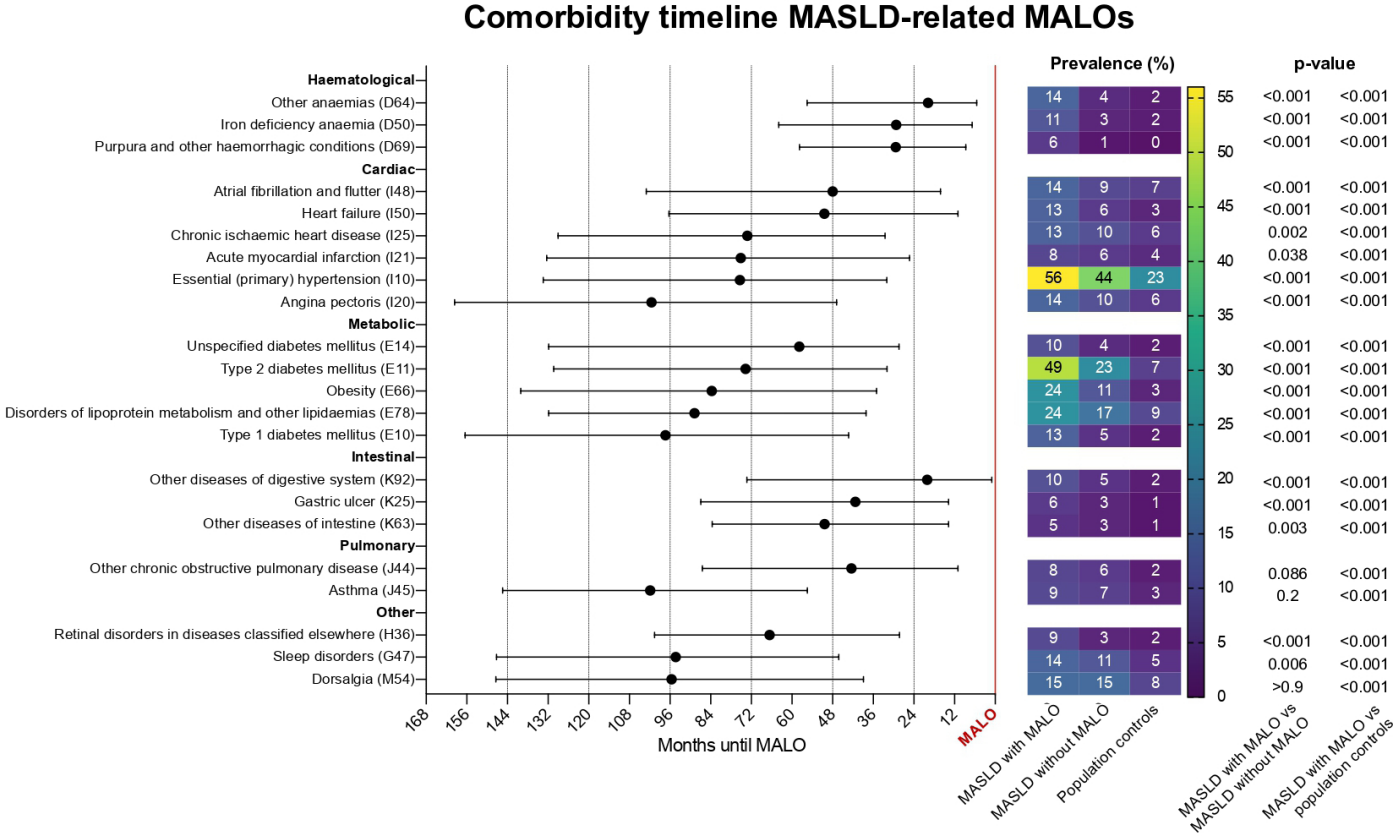
Timeline showing median times of comorbidities preceding a diagnosis of MASLD-related MALO. The prevalences of the identified comorbidities at the time of the MASLD-related MALO are shown in the right panel with a comparison to matched patients with MASLD without MALO and population controls. MALO, major adverse liver outcome; MASLD, metabolic dysfunction-associated steatotic liver disease.

Median times between the first diagnoses of the comorbidities in the cardiometabolic subcluster and first MASLD-related MALO were determined (figure 4, values in online supplemental table 4). Haematological conditions generally occurred the closest to MALOs, among which were ‘other anaemias’ (20 months; IQR=5–56) and ‘iron deficiency anaemia’ (29 months; IQR=7–64). Cardiac diseases were present earlier in the disease course towards a MALO, with median times to event of 48 (IQR=16–103) and 50 (IQR=11–96) months for ‘atrial fibrillation and flutter’ and ‘heart failure’, and 75 (IQR=25–132) and 101 (IQR=47–160) months for ‘acute myocardial infarction’ and ‘angina pectoris’, respectively.

Specific metabolic conditions preceding MALOs were ‘T2DM’ (74 months; IQR=32–130), ‘obesity’ (84 months; IQR=35–140), ‘disorders of lipoprotein metabolism and other lipidaemias’ (89 months; IQR=38–132) and ‘type 1 diabetes mellitus’ (97 months; IQR=43–157). Among other identified comorbidities were ‘gastric ulcer’ (41 months; IQR=14–87), ‘retinal disorders in diseases classified elsewhere’ (67 months; IQR=28–101) and ‘sleep disorders’ (94 months; IQR=46–147).

### Patterns of comorbidities between the groups

Prevalences of specific comorbidities that proportionally defined the largest part of the comorbidity cluster in patients with a MASLD-related MALO were 56% for ‘essential hypertension’, 49% for ‘T2DM’, 24% for ‘disorders of lipoprotein metabolism and other lipidaemias’ and 24% for ‘obesity’. Corresponding numbers in patients with MASLD without MALO and population controls were 44% and 23% for ‘essential hypertension’, 23% and 7% for ‘T2DM’, 17% and 9% for ‘disorders of lipoprotein metabolism and other lipidaemias’ and 11% and 3% for ‘obesity’ (figure 4, all numbers in online supplemental figure 6).

Most patients with MASLD-related MALO and ‘T2DM’ also had ‘essential hypertension’ (36% of the study sample), while 32% had no previous diagnosis of ‘T2DM’ or ‘essential hypertension’ (online supplemental table 5).

Comorbidities beyond cardiometabolic risk factors, with corresponding numbers for individuals with MASLD without MALO and population controls, were ‘sleep disorders’ (which includes ‘sleep apnoea’) (14%, 11% and 5%), ‘heart failure’ (13%, 6% and 3%), ‘atrial fibrillation and flutter’ (14%, 9% and 7%), ‘acute myocardial infarction’ (8%, 6% and 4%) and ‘angina pectoris’ (14%, 10% and 6%) (figure 4). Cardiac disease (ie*,* ischaemic heart disease, atrial fibrillation/flutter or heart failure) was present in 31% of patients with MALO and occurred in ~2/3 of cases in concurrence with T2DM (online supplemental table 5).

Among 12 clinically relevant conditions from the cardiometabolic cluster (specified in online supplemental table 5), patients with MASLD-related MALO had a median of 2 comorbidities (IQR=1–4), which was higher compared with individuals with MASLD without MALO (median 1; IQR=0–2, p<0.001) and population controls (median 0; IQR=0–1, p<0.001). Corresponding mean numbers were 3 (SD=2), 2 (SD=2) and 1 (SD=1) comorbidities. Patients with MASLD-related MALO had two or more of these comorbidities in 63% of the cases, while 19% had no coding for one of these conditions prior to MALO (online supplemental figure 7).

## Discussion

MALOs pose a substantial health risk to patients with or at risk for MASLD and are the primary incentive to screen for advanced fibrosis, as suggested by international guidelines.^6^ Individuals at particularly high risk for developing MASLD-related advanced fibrosis include people with T2DM, of whom up to 70% have MASLD and up to 19% may have advanced fibrosis.^7^ Although stepwise algorithms for finding patients with MASLD-related advanced fibrosis have been introduced in clinical guidelines to prevent progression to cirrhosis and its complications,^6^ a recent meta-analysis showed that 3.3% of patients in a general practice setting have cirrhosis,^8^ indicating the need for additional case finding strategies and better understanding of their natural history prior to cirrhosis development. Here, ischaemic heart disease, atrial fibrillation/flutter, heart failure and sleep disorders, in concurrence with cardiometabolic risk factors—particularly T2DM and essential hypertension—were identified as potentially relevant cues for performing liver-directed investigations or intensified monitoring of MASLD progression, with clinically relevant differences in prevalence between patients with MASLD-related MALO and MASLD without MALO, and even lower occurrence in general population controls. Different types of anaemias also preceded MALOs, although a time window of ~2 years may be too short to take meaningful preventive measures.^5 6^ Further, anaemia in patients with cirrhosis may have been caused by chronic blood loss related to portal hypertension and hypersplenism,^16^ suggesting that these patients already had cirrhosis at the time of anaemia diagnosis.

The coaggregation of MASLD with cardiovascular disease is well known,^17^ and MASLD increases the risk for both developing atrial fibrillation (HR 1.20)^18^ and heart failure (HR 1.50),^11^ which could explain these findings. Overt cardiovascular disease in patients with MASLD could hence reflect long-lasting systemic metabolic stress, with impact on both liver and cardiovascular health.^19^

Mechanistically, systemic inflammation could link progressive MASLD with atrial fibrillation,^20^ while congestive hepatopathy may contribute to fibrosis progression in patients with heart failure.^21^ Case finding of patients with MASLD at high risk for developing MALOs could, thus, occur by targeting individuals with multiple cardiometabolic risk factors in conjunction with cardiac disease, which will ultimately require a multidisciplinary approach.^2 21^ Other relevant comorbidities in the cluster of patients with MASLD-related MALO were obesity and sleep disorders. These factors can be explained by the disease-modifying effect of obesity on MASLD through metabolic inflammation and free fatty acid fluxes^19^ and the occurrence of MASLD in ~75% of people living with obesity,^22^ while obesity is a risk factor for developing sleep apnoea.^23^ In addition, the internal circadian rhythm regulates nutrient homeostasis and lipid metabolism, which can get disturbed following sleep problems.^24^

Recently, a potential new strategy for case finding of people living with advanced fibrosis was explored in which liver vibration-controlled transient elastography was offered to patients with T2DM (n=1301) undergoing routine retina scanning. Liver examination was accepted by 77% of the study participants and showed that only 2.9% had liver stiffness values exceeding 12 kPa, which is suggestive of advanced fibrosis, with around 50% of patients having steatosis.^25^ Case finding of individuals at high risk for developing MASLD-related MALOs could, therefore, potentially be more effective in patients with T2DM who also have other conditions related to long-lasting metabolic dysregulation, including hypertension, ischaemic heart disease, heart failure and atrial fibrillation.^17 19^ Indeed, patients with T2DM have higher risks for MALOs if they have additional components of the metabolic syndrome, especially hypertension.^26^

Since the individuals with MASLD who developed a MALO were multimorbid, early identification should likely include lifestyle modifications and drugs targeting broad metabolic mechanisms. Since GLP-1 receptor agonists reduce the risk of MALOs in patients with chronic liver disease and T2DM,^27^ while also reducing the risk of major adverse cardiovascular events,^28^ their use can be positioned at the nexus of both progressive MASLD and cardiac disease. Such holistic strategies may not only mitigate the risk for developing other metabolic sequelae related to MASLD^29^ but also the excess mortality, which in particular originates from non-HCC cancer (15-year cumulative incidence of 7.3%) and cardiovascular disease (15-year cumulative incidence of 7.2%).^30^

The results should be interpreted in the context of the study limitations. First, the initial ICD-10 code screening was based on ICD-10 categories, which sometimes include heterogeneous diseases and conditions, and specific diseases may be mismatched between examined groups. Second, the earlier an individual was included in the study, the shorter the period was to ascertain comorbidities defined by ICD-10 codes, although most study subjects were included in the last years of the study period. Third, diseases considered as less severe can be undercoded, such as is the case for obesity, although the positive predictive value for most diagnoses ranges between 85% and 95% in the Swedish inpatient register.^31^ In line with this, coding for ascites as a component of the case definition of decompensated cirrhosis could have occurred in the context of heart failure or other diseases, although all patients had concomitant MASLD or MASH.^32^ Further, there does not exist an ICD-10 code for metabolic dysfunction and alcohol-related liver disease (MetALD), which could have resulted in coding for MASLD in patients consuming significant amounts of alcohol but not reaching the criteria of alcohol-related liver disease.^33^ Also, diagnostic rates for certain diseases could have changed throughout the years because of changes in clinical guidelines, approval of new drugs and changing reimbursement policies. Finally, the observational study design did not allow ascertaining the potential added value of case finding using the identified comorbidities in conjunction with current strategies and did neither prove any causal relationship. On the contrary, the nationwide coverage of the study permitted a sample size of nearly 1000 patients with a MASLD-related MALO, which in combination with the untargeted ICD-10 categories screening resulted in a reliable cluster analysis. Further, the strict exclusion criteria for both other liver diseases and conditions that predispose to certain other liver diseases reduced the risk of misclassification bias, although undisclosed high alcohol consumption cannot be ruled out. In addition, it has been shown that historical ICD coding for non-alcoholic fatty liver disease (NAFLD) corresponds well to MASLD when considering different diagnostic criteria.^34^ Finally, we could not account for socioeconomic differences and genetic background, but the external validity of the findings is considered as high within a Western setting because of the utilisation of an up-to-date database reflecting the general population in Sweden.

## Conclusion

Persons experiencing a MASLD-related MALO are frequently multimorbid, with around nine other diseases. Cardiac disease, including ischaemic heart disease, heart failure and atrial arrhythmias, and sleep disorders, often precede the development of a MASLD-related MALO with a time window of 4–8 years and could, hence, be useful for case finding or intensified monitoring of advanced MASLD. Future studies are needed to clarify the clinical utility of employing these conditions in a case finding strategy for preventing MALO development in patients with cardiometabolic risk factors.

## Supplementary material

10.1136/bmjph-2025-003322 online supplemental file 1

10.1136/bmjph-2025-003322 online supplemental file 2

10.1136/bmjph-2025-003322 online supplemental file 3

10.1136/bmjph-2025-003322 online supplemental file 5

## Data Availability

Data may be obtained from a third party and are not publicly available.
