# Supplementary figures and images for: Temporal comorbidity patterns preceding MASLD-related major adverse liver outcomes: a nationwide population-based case–control study in Sweden
Source: BMJ Public Health. 2025 Nov 13;3(2):e003322. doi: 10.1136/bmjph-2025-003322 (PMC12625918; doi:10.1136/bmjph-2025-003322)

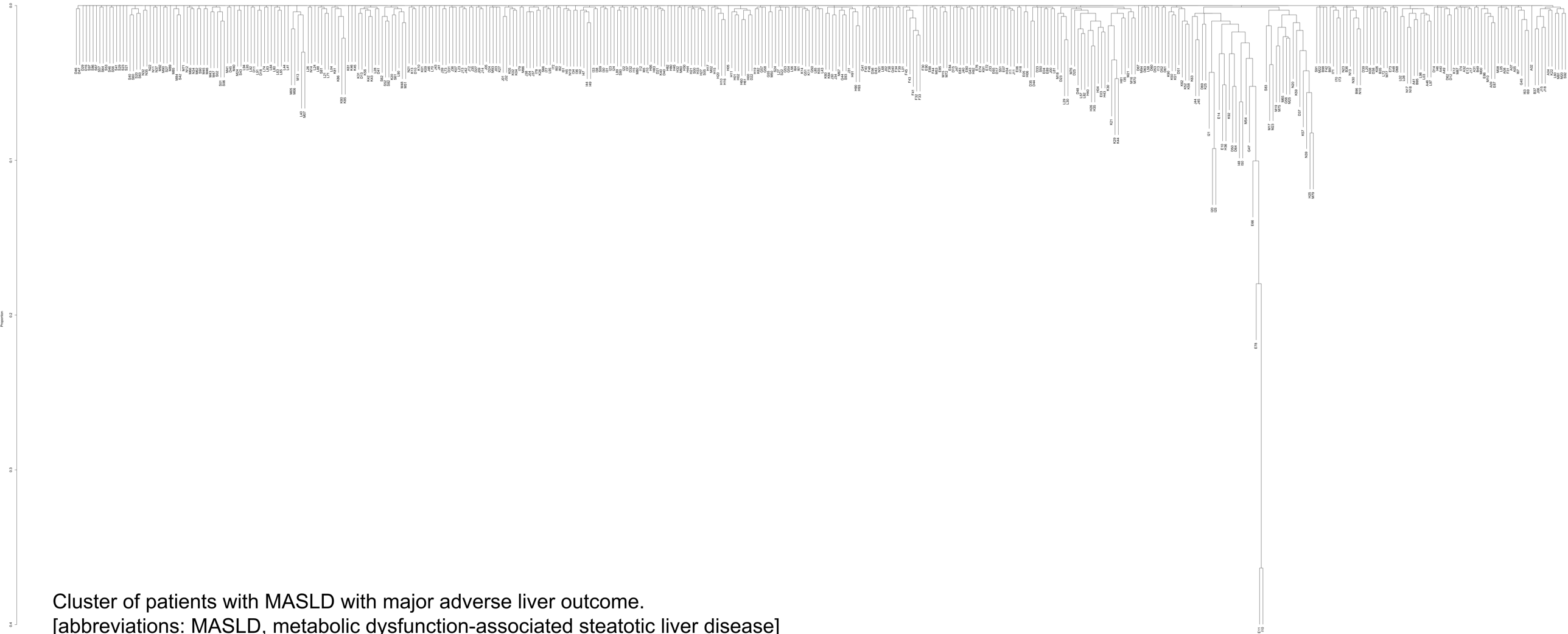

Supplement: online supplemental file 1 [file bmjph-3-2-s001.pdf]

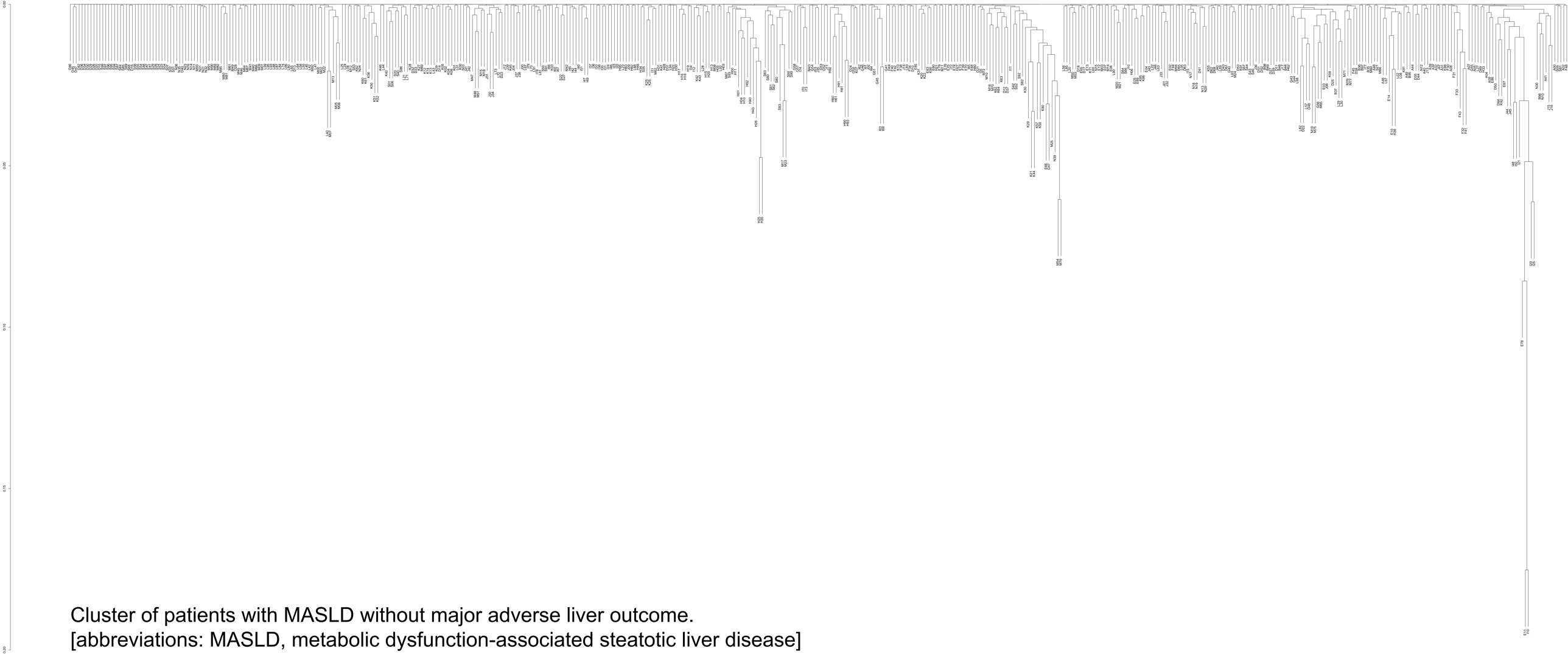

Supplement: online supplemental file 2 [file bmjph-3-2-s002.pdf]

Cluster of population controls.

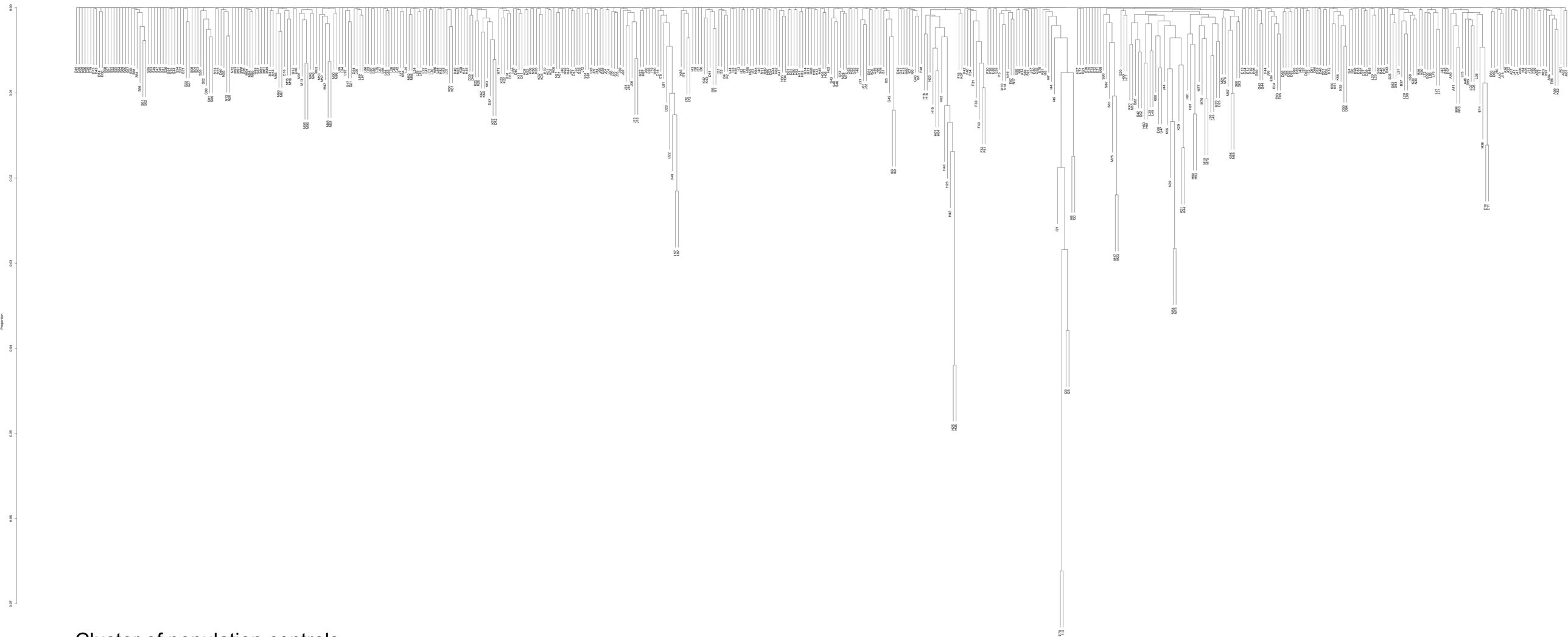

Supplement: online supplemental file 3 [file bmjph-3-2-s003.pdf]
