# Supplementary material for: Temporal comorbidity patterns preceding MASLD-related major adverse liver outcomes: a nationwide population-based case–control study in Sweden
Source: BMJ Public Health. 2025 Nov 13;3(2):e003322. doi: 10.1136/bmjph-2025-003322 (PMC12625918; doi:10.1136/bmjph-2025-003322)
Supplement: online supplemental file 5 [file bmjph-3-2-s004.pdf]

Supplementary material

Temporal comorbidity patterns preceding MASLD-related major adverse liver outcomes: a nationwide population-based case-control study in Sweden

Joost Boeckmans, Linnea Widman, Rickard Strandberg, Ying Shang, Axel Wester, and Hannes Hagström

Contents

Supplementary figures……………. p. 2

Supplementary tables……..………. p. 9

Supplementary figures

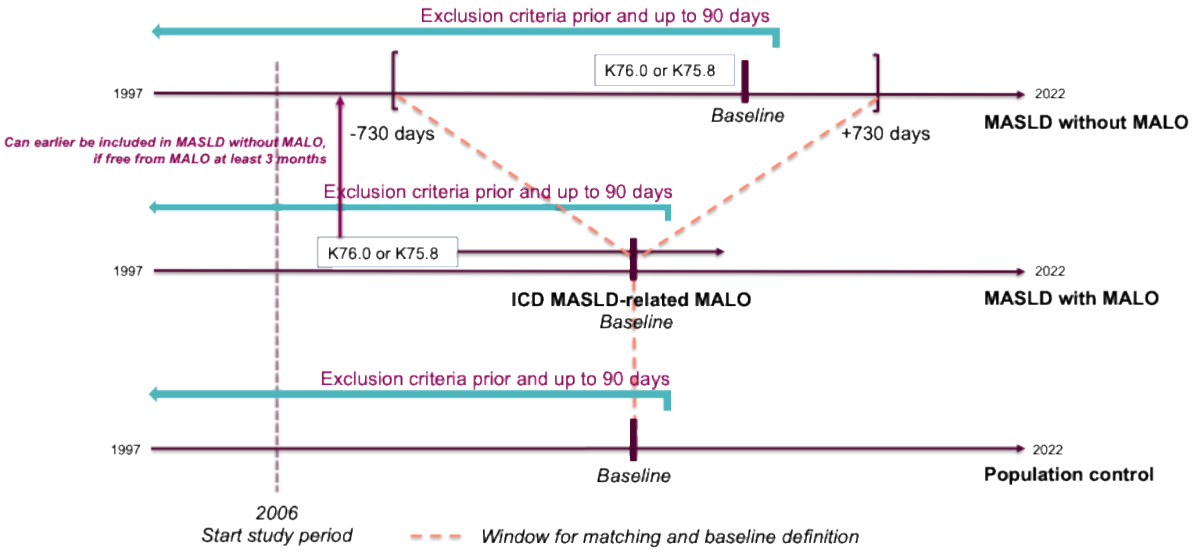

Supplementary figure 1: Study design. [abbreviation: MALO, major adverse liver outcome; MASLD, metabolic dysfunction-associated steatotic liver disease]

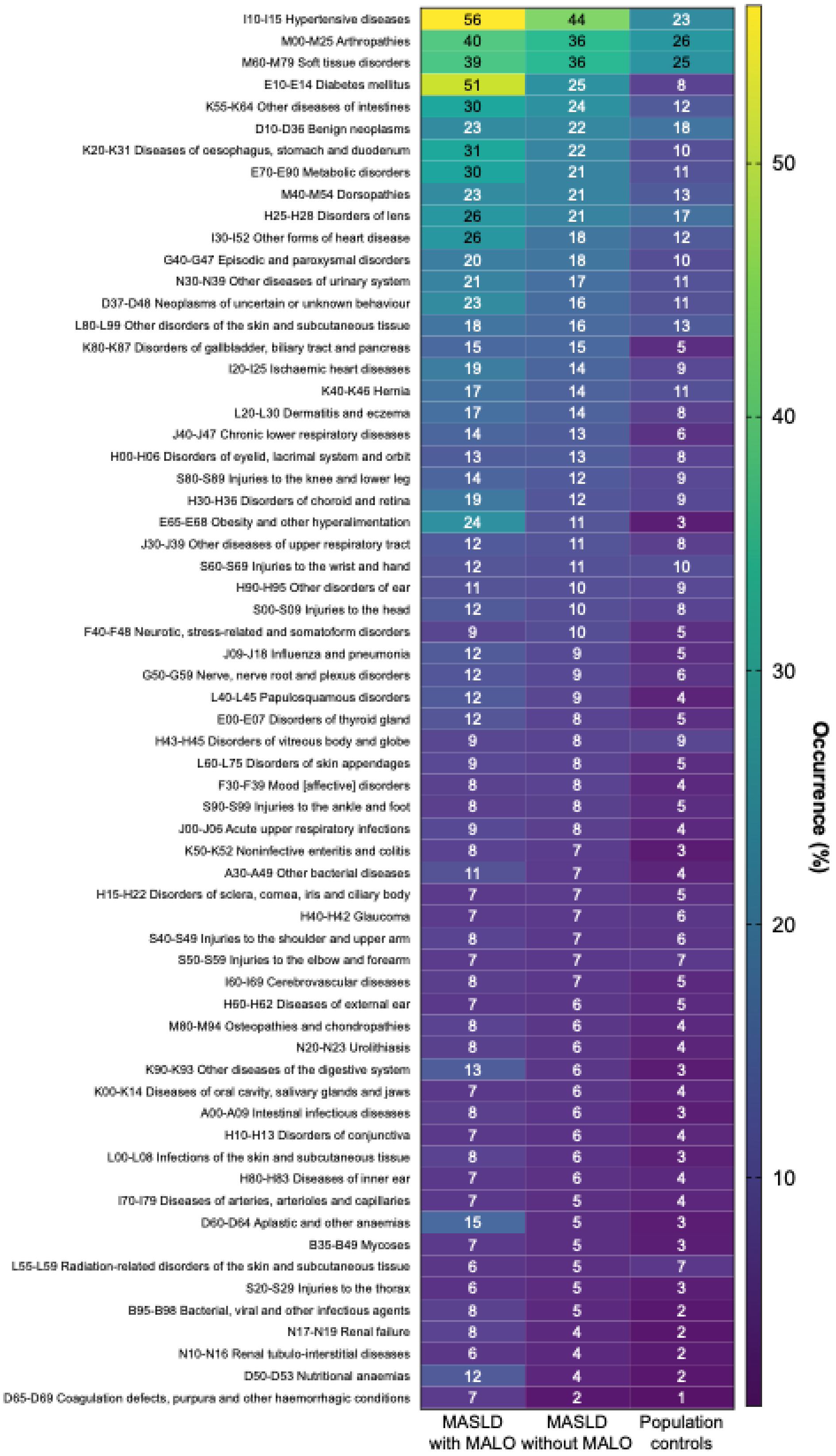

Supplementary figure 2: Occurrence of ICD-10 categories in patients with MASLD-related MALO and matched individuals with MASLD without MALO and population controls, sorted by MASLD without MALO. A cutoff of 5% occurrence was applied to the ICD-10 categories in patients with MASLD-related MALO. [abbreviations: ICD, International Classification of Diseases; MALO, major adverse liver outcome; MASLD, metabolic dysfunction-associated steatotic liver disease]

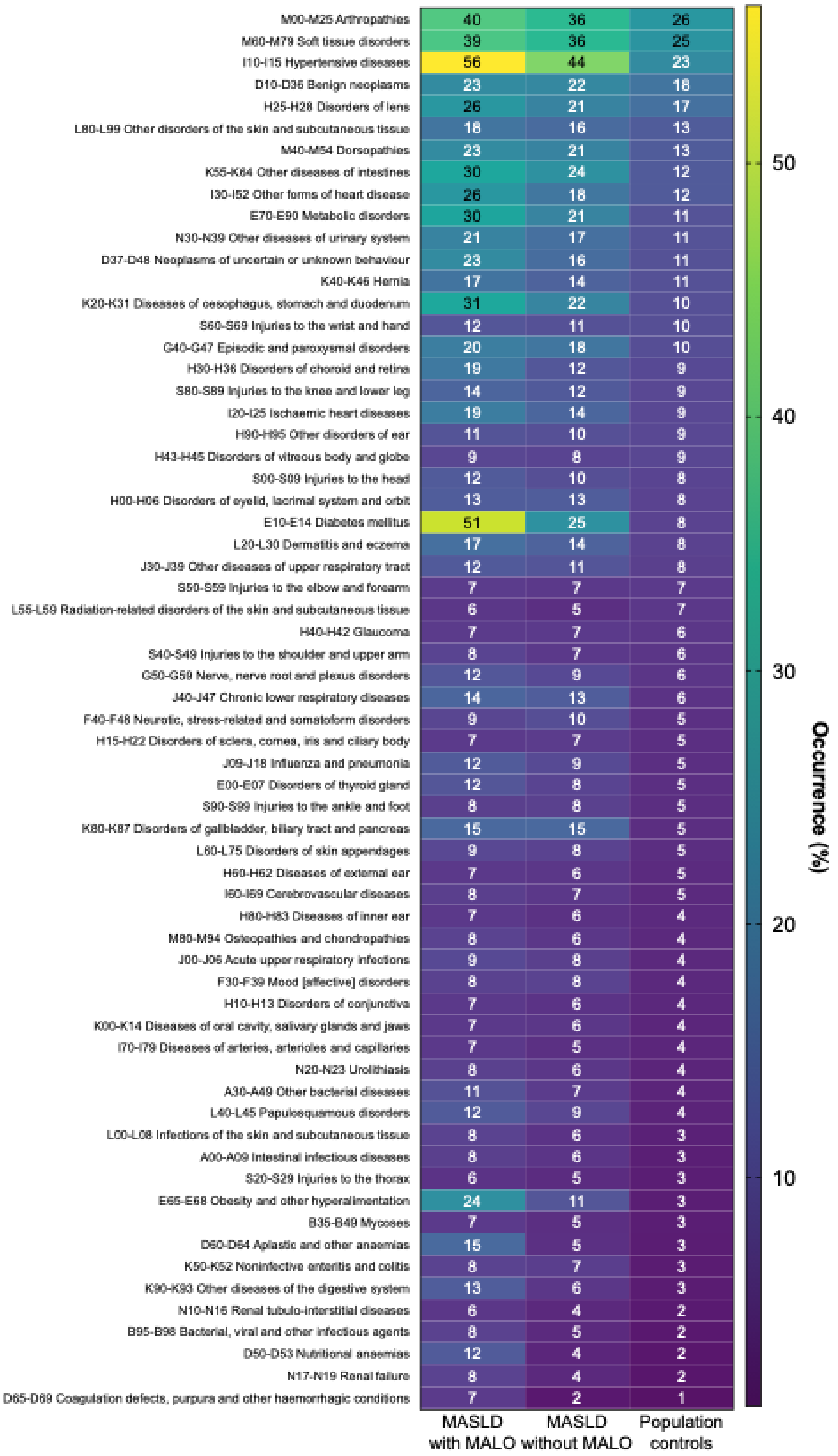

Supplementary figure 3: Occurrence of ICD-10 categories in patients with MASLD-related MALO and matched individuals with MASLD without MALO and population controls, sorted by population controls. A cutoff of 5% occurrence was applied to the ICD-10 categories in patients with MASLD-related MALO. [abbreviations: ICD, International Classification of Diseases; MALO, major adverse liver outcome; MASLD, metabolic dysfunction-associated steatotic liver disease]

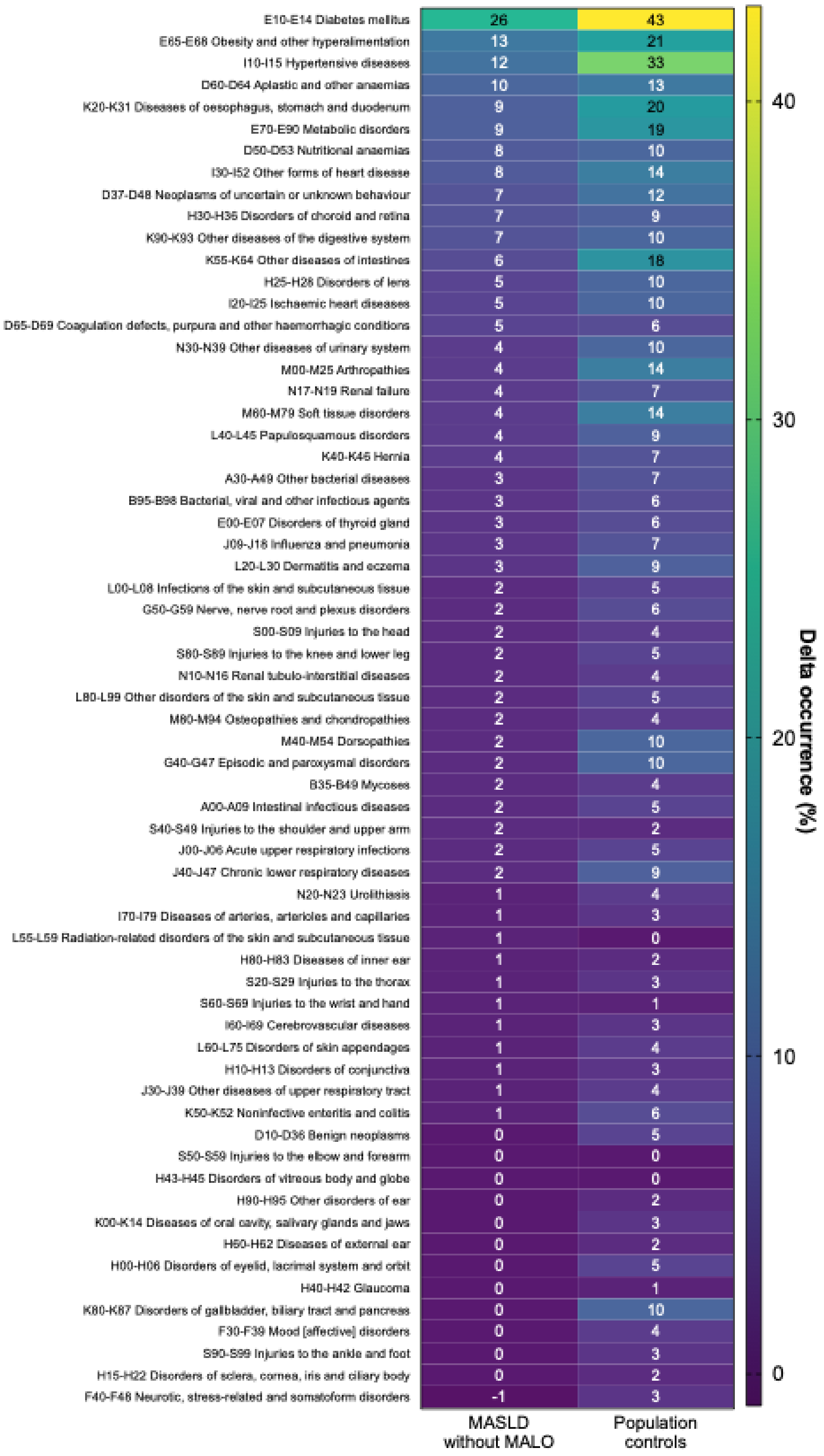

Supplementary figure 4: Difference in occurrence of ICD-10 categories in patients with MASLD-related MALO compared with matched individuals with MASLD without MALO and population controls, sorted by MASLD without MALO. [abbreviations: ICD, International Classification of Diseases; MALO, major adverse liver outcome; MASLD, metabolic dysfunction-associated steatotic liver disease]

A. **Comorbidity cluster MASLD without MALO, including type 2 diabetes mellitus**

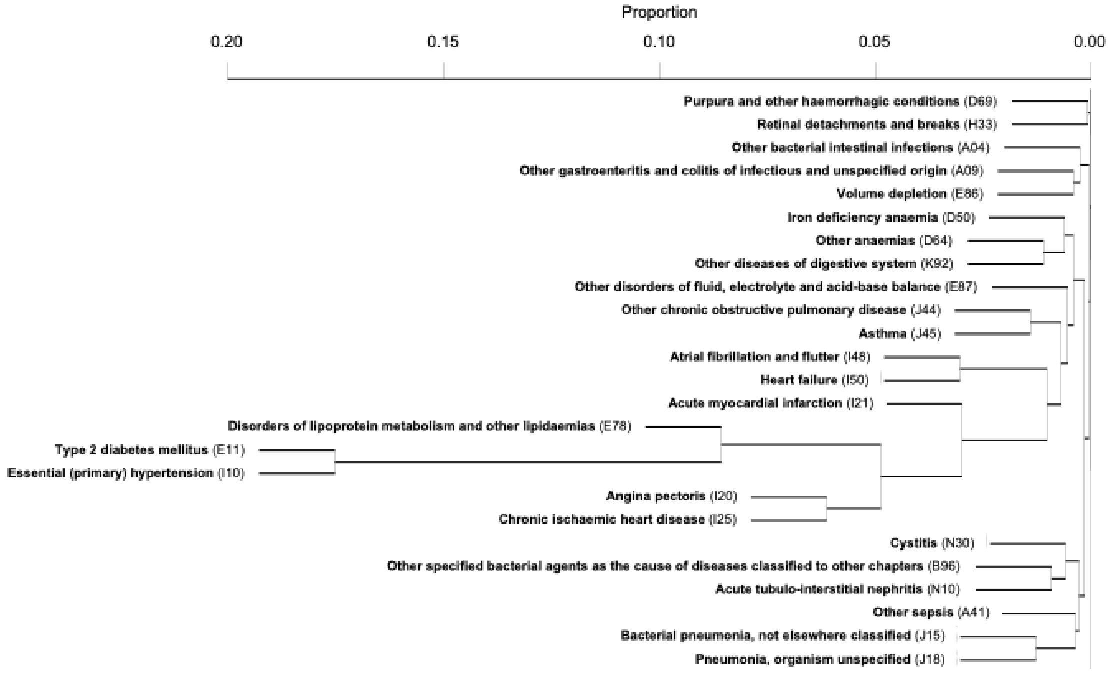

B. **Comorbidity cluster general population, including type 2 diabetes mellitus**

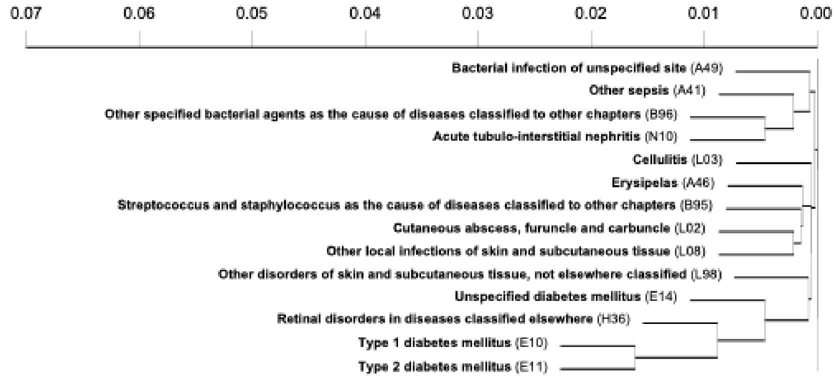

Supplementary figure 5: Comorbidity clusters of individuals with MASLD without MALO (A) and population controls (B), including type 2 diabetes mellitus. [abbreviations: MALO, major adverse liver outcome; MASLD, metabolic dysfunction-associated steatotic liver disease]

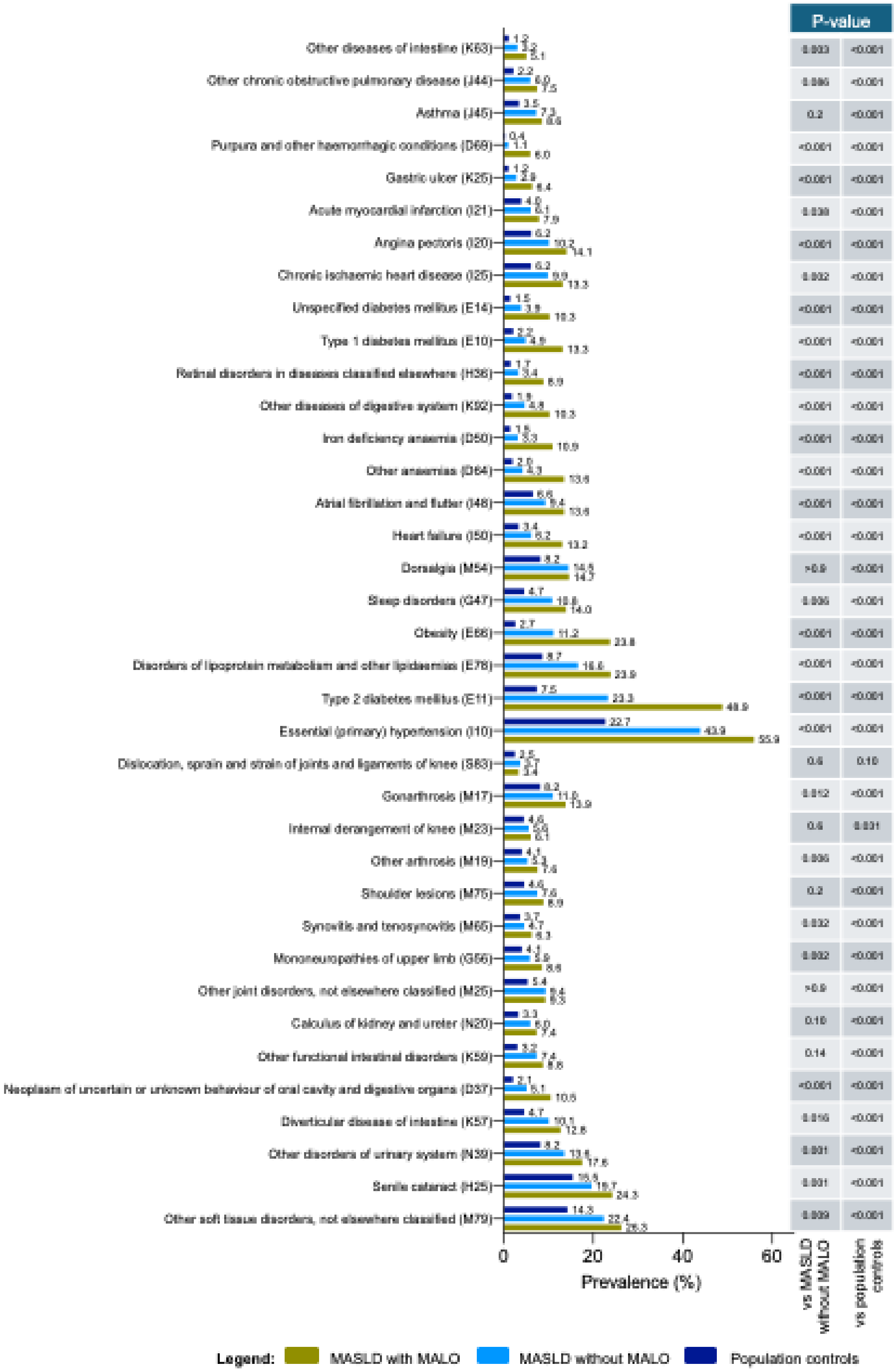

Supplementary figure 6: Prevalence of identified comorbidities relevant to MASLD-related MALO, compared with individuals having MASLD without MALO and population controls. [abbreviations: MALO, major adverse liver outcome; MASLD, metabolic dysfunction-associated steatotic liver disease]

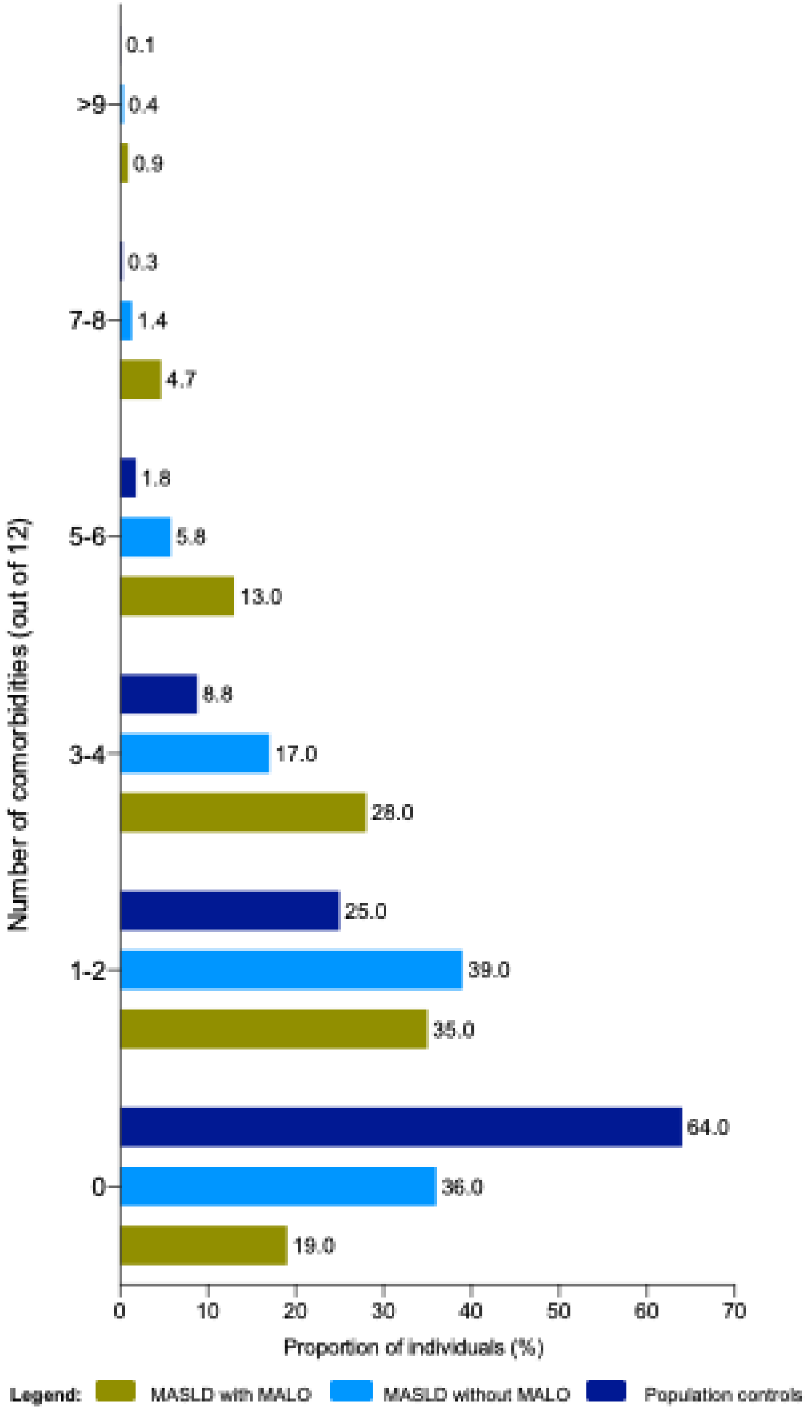

Supplementary figure 7: Number of comorbidities in patients MASLD-related MALO, individuals with MASLD without MALO, and population controls. Comorbidities consisted of 12 potential conditions: (Iron deficiency anaemia (D50) or Other anaemias (D64)), (Type 1 diabetes mellitus (E10) or Type 2 diabetes mellitus (E11) or Unspecified diabetes mellitus (E14)), (Angina pectoris (I20) or Acute myocardial infarction (I21) or Chronic ischaemic heart disease (I25)), Obesity (E66), Disorders of lipoprotein metabolism and other lipidaemias (E78), Sleep disorders (G47), Retinal disorders in diseases classified elsewhere (H36), Essential (primary) hypertension (I10), Atrial fibrillation, and flutter (I48), Heart failure (I50), Other chronic obstructive pulmonary disease (J44), and Asthma (J45)). [abbreviations: MALO, major adverse liver outcome; MASLD, metabolic dysfunction-associated steatotic liver disease].

Supplementary tables

Supplementary table 1. ICD-10 codes used to define MASLD-related MALOs. [abbreviations: ATC, anatomical therapeutic chemical; HCC, hepatocellular carcinoma; ICD, International Classification of Diseases; MALO, major adverse liver outcome; MASLD, metabolic dysfunction-associated steatotic liver disease]

	Disease	ICD-10
Compensated MASLD-cirrhosis	Liver cirrhosis, unspecified	K74.6 without ATC A07AA11 (rifaximin)
	Oesophageal varices, not bleeding	I85.9, I98.2
	Gastric varices, not bleeding	I86.4
Decompensated MASLD-cirrhosis	Oesophageal varices, bleeding	I85.0, I98.3
	Ascites	R18
	Hepatic encephalopathy	K74.6 with ATC A07AA11 (rifaximin)
	Hepatorenal syndrome	K76.7
	Portal hypertension	K76.6
HCC	Hepatocellular carcinoma, Liver cancer, unspecified	C22.0, C22.9
Liver transplantation	End-stage MASLD requiring liver transplantation	Z94.4, OP: JJC00, JJC10, JJC20, DJ005, DJ006, JJC30, JJC40

Supplementary table 2. ICD-10 codes for exclusions.
(National Patient Register, in- and out-patients)

Diagnosis	ICD-10
**Other liver diseases**	
Alcohol-related liver disease	K70
Viral hepatitis	B16-B19
Autoimmune liver disease (auto-immune hepatitis, primary biliary cholangitis, primary sclerosing cholangitis)	K83, K74.3, K75.4
Hemochromatosis	E83.1
Wilson's disease	E83.0B
Alfa-1 antitrypsin-deficiency	E88.0A, E88.0B
Budd-Chiari syndrome	I82.0, K76.5
Chronic hepatitis, unspecified	K73.9, K73.2
Secondary or unspecified biliary cirrhosis	K74.4, K74.5
**Alcohol or drug abuse**	
Alcohol use disorder	F10
Somatic consequences of alcohol (except ALD)	E24.4, G62.1, I42.6, K29.2, G31.2, G72.1, K85.2, K86.0, T51.0, T51.9, Y57.3, X65, Z71.4, Z72.1
Drug use disorders (except nicotine/caffeine)	F11-14, F16, F18, F19

Supplementary table 3. ICD-10 codes for clustering.
[abbreviation: ICD, International Classification of Diseases]

No.	ICD-10 category	Corresponding ICD-10 codes
1	A00-A09 Intestinal infectious diseases	A00 A01 A02 A03 A04 A05 A06 A07 A08 A09
2	A30-A49 Other bacterial diseases	A30 A31 A32 A33 A34 A35 A36 A37 A38 A39 A40 A41 A42 A43 A44 A46 A48 A49
3	B35-B49 Mycoses	B35 B36 B37 B38 B39 B40 B41 B42 B43 B44 B45 B46 B47 B48 B49
4	B95-B98 Bacterial, viral and other infectious agents	B95 B96 B97 B98
5	D10-D36 Benign neoplasms	D10 D11 D12 D13 D14 D15 D16 D17 D18 D19 D20 D21 D22 D23 D24 D25 D26 D27 D28 D29 D30 D31 D32 D33 D34 D35 D36
6	D37-D48 Neoplasms of uncertain or unknown behaviour	D37 D38 D39 D40 D41 D42 D43 D44 D45 D46 D47 D48
7	D50-D53 Nutritional anaemias	D50 D51 D52 D53
8	D60-D64 Aplastic and other anaemias	D60 D61 D62 D63 D64
9	D65-D69 Coagulation defects, purpura and other haemorrhagic conditions	D65 D66 D67 D68 D69
10	E00-E07 Disorders of thyroid gland	E00 E01 E02 E03 E04 E05 E06 E07
11	E10-E14 Diabetes mellitus	E10 E11 E12 E13 E14
12	E65-E68 Obesity and other hyperalimentation	E65 E66 E67 E68
13	E70-E90 Metabolic disorders	E70 E71 E72 E73 E74 E75 E76 E77 E78 E79 E80 E83 E8 E85 E86 E87 E88 E89 E90
14	F30-F39 Mood [affective] disorders	F30 F31 F32 F33 F34 F38 F39
15	F40-F48 Neurotic, stress-related and somatoform disorders	F40 F41 F42 F43 F44 F45 F48
16	G40-G47 Episodic and paroxysmal disorders	G40 G41 G43 G44 G45 G46 G47
17	G50-G59 Nerve, nerve root and plexus disorders	G50 G51 G52 G53 G54 G55 G56 G57 G58 G59
18	H00-H06 Disorders of eyelid, lacrimal system and orbit	H00 H01 H02 H03 H04 H05 H06
19	H10-H13 Disorders of conjunctiva	H10 H11 H13
20	H15-H22 Disorders of sclera, cornea, iris and ciliary body	H15 H16 H17 H18 H19 H20 H21 H22
21	H25-H28 Disorders of lens	H25 H26 H27 H28
22	H30-H36 Disorders of choroid and retina	H30 H31 H32 H33 H34 H35 H36
23	H40-H42 Glaucoma	H40 H42
24	H43-H45 Disorders of vitreous body and globe	H43 H44 H45
25	H60-H62 Diseases of external ear	H60 H61 H62
26	H80-H83 Diseases of inner ear	H80 H81 H82 H83
27	H90-H95 Other disorders of ear	H90 H91 H92 H93 H94 H95
28	I10-I15 Hypertensive diseases	I10 I11 I12 I13 I15
29	I20-I25 Ischaemic heart diseases	I20 I21 I22 I23 I24 I25
30	I30-I52 Other forms of heart disease	I30 I31 I32 I33 I34 I35 I36 I37 I38 I39 I40 I41 I42 I43 I44 I45 I46 I47 I48 I49 I50 I51 I52
31	I60-I69 Cerebrovascular diseases	I60 I61 I62 I63 I64 I65 I66 I67 I68 I69
32	I70-I79 Diseases of arteries, arterioles and capillaries	I70 I71 I72 I73 I74 I77 I78 I79
33	J00-J06 Acute upper respiratory infections	J00 J01 J02 J03 J04 J05 J06
34	J09-J18 Influenza and pneumonia	J09 J10 J11 J12 J13 J14 J15 J16 J17 J18
35	J30-J39 Other diseases of upper respiratory tract	J30 J31 J32 J33 J34 J35 J36 J37 J38 J39
36	J40-J47 Chronic lower respiratory diseases	J40 J41 J42 J43 J44 J45 J46 J47
37	K00-K14 Diseases of oral cavity, salivary glands and jaws	K00 K01 K02 K03 K04 K05 K06 K07 K08 K09 K10 K11 K12 K13 K14
38	K20-K31 Diseases of oesophagus, stomach and duodenum	K20 K21 K22 K23 K25 K26 K27 K28 K29 K30 K31
39	K40-K46 Hernia	K40 K41 K42 K43 K44 K45 K46
40	K50-K52 Noninfective enteritis and colitis	K50 K51 K52
41	K55-K64 Other diseases of intestines	K55 K56 K57 K58 K59 K60 K61 K62 K63 K64
42	K80-K87 Disorders of gallbladder, biliary tract and pancreas	K80 K81 K82 K83 K85 K86 K87
43	K90-K93 Other diseases of the digestive system	K90 K91 K92 K93

44	L00-L08 Infections of the skin and subcutaneous tissue	L00 L01 L02 L03 L04 L05 L08
45	L20-L30 Dermatitis and eczema	L20 L21 L22 L23 L24 L25 L26 L27 L28 L29 L30
46	L40-L45 Papulosquamous disorders	L40 L41 L42 L43 L44 L45
47	L55-L59 Radiation-related disorders of the skin and subcutaneous tissue	L55 L56 L57 L58 L59
48	L60-L75 Disorders of skin appendages	L60 L62 L63 L64 L65 L66 L67 L68 L70 L71 L72 L73 L74 L75
49	L80-L99 Other disorders of the skin and subcutaneous tissue	L80 L81 L82 L83 L84 L85 L86 L87 L88 L89 L90 L91 L92 L93 L94 L95 L97 L98 L99
50	M00-M25 Arthropathies	M00 M01 M02 M03 M05 M06 M07 M08 M09 M10 M11 M12 M13 M14 M15 M16 M17 M18 M19 M20 M21 M22 M23 M24 M25
51	M40-M54 Dorsopathies	M40 M41 M42 M43 M45 M46 M47 M48 M49 M50 M51 M53 M54
52	M60-M79 Soft tissue disorders	M60 M61 M62 M63 M65 M66 M67 M68 M70 M71 M72 M73 M75 M76 M77 M79
53	M80-M94 Osteopathies and chondropathies	M80 M81 M82 M83 M84 M85 M86 M87 M88 M89 M90 M9 M92 M93 M94
54	N10-N16 Renal tubulo-interstitial diseases	N10 N11 N12 N13 N14 N15 N16
55	N17-N19 Renal failure	N17 N18 N19
56	N20-N23 Urolithiasis	N20 N21 N22 N23
57	N30-N39 Other diseases of urinary system	N30 N31 N32 N33 N34 N35 N36 N37 N39
58	S00-S09 Injuries to the head	S00 S01 S02 S03 S04 S05 S06 S07 S08 S09
59	S20-S29 Injuries to the thorax	S20 S21 S22 S23 S24 S25 S26 S27 S28 S29
60	S40-S49 Injuries to the shoulder and upper arm	S40 S41 S42 S43 S44 S45 S46 S47 S48 S49
61	S50-S59 Injuries to the elbow and forearm	S50 S51 S52 S53 S54 S55 S56 S57 S58 S59
62	S60-S69 Injuries to the wrist and hand	S60 S61 S62 S63 S64 S65 S66 S67 S68 S69
63	S80-S89 Injuries to the knee and lower leg	S80 S81 S82 S83 S84 S85 S86 S87 S88 S89
64	S90-S99 Injuries to the ankle and foot	S90 S91 S92 S93 S94 S95 S96 S97 S98 S99

Supplementary table 4. Median times (in months) of identified comorbidities until a first MASLD-related MALO. [abbreviations: ICD, International Classification of Diseases; MALO, major adverse liver outcome; MASLD, metabolic dysfunction-associated steatotic liver disease]

Comorbiditiy with ICD-10 code	Median time until MALO (months)	Interquartile range lower boundary	Interquartile range upper boundary
**Haematological**			
Other anaemias (D64)	20	5	56
Iron deficiency anaemia (D50)	29	7	64
Purpura and other haemorrhagic conditions (D69)	29	9	58
**Cardiac**			
Atrial fibrillation and flutter (I48)	48	16	103
Heart failure (I50)	50	11	96
Chronic ischaemic heart disease (I25)	73	32	129
Acute myocardial infarction (I21)	75	25	132
Essential (primary) hypertension (I10)	75	32	133
Angina pectoris (I20)	101	47	160
**Metabolic**			
Unspecified diabetes mellitus (E14)	58	28	132
Type 2 diabetes mellitus (E11)	74	32	130
Obesity (E66)	84	35	140
Disorders of lipoprotein metabolism and other lipidaemias (E78)	89	38	132
Type 1 diabetes mellitus (E10)	97	43	157
**Intestinal**			
Other diseases of digestive system (K92)	20	1	73
Gastric ulcer (K25)	41	14	87
Other diseases of intestine (K63)	50	14	84
**Pulmonary**			
Other chronic obstructive pulmonary disease (J44)	42	11	86
Asthma (J45)	102	55	145
**Other**			
Retinal disorders in diseases classified elsewhere (H36)	67	28	101
Sleep disorders (G47)	94	46	147
Dorsalgia (M54)	96	39	147

Supplementary table 5. Concurrence of comorbidities identified from cardiometabolic cluster in patients with MASLD-related MALO.

*Cardiac disease was defined as angina pectoris (I20) or acute myocardial infarction (I21) or chronic ischaemic heart disease (I25) or atrial fibrillation and flutter (I48) or heart failure (I50).

**out of clinically relevant and unrelated conditions (Iron deficiency anaemia (D50) or Other anaemias (D64)), (Type 1 diabetes mellitus (E10) or Type 2 diabetes mellitus (E11) or Unspecified diabetes mellitus (E14)), (Angina pectoris (I20) or Acute myocardial infarction (I21) or Chronic ischaemic heart disease (I25)), Obesity (E66), Disorders of lipoprotein metabolism and other lipidaemias (E78), Sleep disorders (G47), Retinal disorders in diseases classified elsewhere (H36), Essential (primary) hypertension (I10), Atrial fibrillation, and flutter (I48), Heart failure (I50), Other chronic obstructive pulmonary disease (J44), and Asthma (J45)). [abbreviations: MALO, major adverse liver outcome; MASLD, metabolic dysfunction-associated steatotic liver disease]

Parameter	MASLD with MALO (N = 996)	MASLD without MALO (N=3921)	Population controls (N=9056)
**Concurrence of comorbidities from cardiometabolic cluster***			
Type 2 diabetes (E11) without essential hypertension (I10)	125 (13%)	228 (5.8%)	206 (2.3%)
Essential hypertension (I10) without type 2 diabetes (E11)	195 (20%)	1036 (26%)	1586 (18%)
No type 2 diabetes (E11) and no essential hypertension (I10)	314 (32%)	1970 (50%)	6791 (75%)
Both type 2 diabetes (E11) and essential hypertension (I10)	362 (36%)	687 (18%)	473 (5.2%)
Cardiac disease	306 (31%)	877 (22%)	1302 (14%)
Type 2 diabetes (E11) with cardiac disease	197 (20%)	363 (9.3%)	281 (3.1%)
Cardiac disease without type 2 diabetes (E11)	109 (11%)	514 (13%)	1021 (11%)
Sleep disorder (G47)	139 (14%)	425 (11%)	422 (4.7%)
Type 2 diabetes (E11) with sleep disorder (G47)	86 (8.6%)	153 (3.9%)	57 (0.6%)
Sleep disorder (G47) without type 2 diabetes (E11)	53 (5.3%)	272 (6.9%)	365 (4.0%)
**Number of clinically relevant comorbidities from cardiometabolic cluster****			
Median number of comorbidities	2 (1-4)	1 (0-2)	0 (0-1)
2 or more comorbidities	630 (63%)	1654 (42%)	1835 (20%)
